# Mr. Marshall's Medical Topography of the Interior of Ceylon

**Published:** 1822-06-01

**Authors:** 


*Notes on the Medical Topography of the Interior of Ceylon; and on the Health of the Troops employed in the Kandyan Provinces, during the Years* 1815, 1816, 1817, 1818, 1819, *and* 1820: *with brief Remarks on the prevailing Diseases.* By Henry Marshall, Surgeon to the Forces. Octavo, pp. 228. London, 1821.

No nation ever possessed equal disposition and equal power with the English, to investigate what was useful or curious in foreign countries. Our arms or our arts are distributed over the whole surface of the globe. Well might we say—"Quæ regio in terris nostri non plena laboris?"—and well might we smile at the celebrated but now Lilliputian wanderings of Ulysses and Æneas. What were the voyages of Hanno, Nearchus, and Columbus, compared with those of modern navigators? The medical officers, too, of our fleets and armies have contributed their share in raising the reputation of their native island by their scientific researches in foreign seas and lands. The nature of diseases, the influences of climate, and the powers of remedies, have all received great illustration from the labours and the personal sufferings of our brethren attached to the armaments and expeditions of a country so long struggling with the gigantic power of Europe combined in arms against her. The extent of our colonies still offers, even in profound peace, an ample field for etiological, pathological, and therapeutical investigations, especially when aided and stimulated by the heads of naval and military medicine. Mr. Marshall has therefore endeavoured to contribute his mite to our existing stock of information, by pourtraying, or at least sketching, the medical topography, climate, diseases, and natural history of Ceylon, an island with the interior of which we have but lately become much acquainted. The work is dedicated, in terms of respect and gratitude, to Sir James M'Grigor, the distinguished director-general of the Army Medical Department, and makes no pretence to the "graces of composition," the author being anxious "to express himself in terms so as to be clearly understood, and in as few words as the nature of the subject would permit." The author also expresses his obligation to Dr. Theodore Gordon, of the Army Medical Board, for his kind assistance and advice respecting the publication of the present volume.

The work is divided into three parts; the first being on the medical topography of the interior of Ceylon; the second on the health of the troops, &c. and the third on the prevailing diseases of the island. Our limits will not permit us to notice in detail the various subjects briefly discussed in this volume, the whole of which, however, will prove very interesting to those of our brethren, whose destinies may lead them to visit our Indian possessions.

The first chapter presents a very spirited topographical sketch of this far famed island, from which we shall select but a very few particulars. The island is divided naturally into a central or upper, and circumferential or lower country. The extent of the former is as one to eight of the latter. Some of the interior hills rise to upwards of 6000 feet above the level of the sea. The face of this upper country consists of a congeries of detached heights and undulatory swells, interspersed with high and phantastically-peaked mountains, the whole, in general, covered with trees and underwood. The ground is, for the most part, strewed with decaying and decayed vegetables, especially leaves.

"The lower and comparatively flat portion of the island, is of very considerable extent: like the elevated terrace, it is almost completely covered with jungle and forest-trees. The scene is here extremely dull and uniform. There is nothing to diversify the prospect. One forest succeeds another, divided only by narrow strips of unwooded surface, which are for the most part uncultivated. The traveller is frequently in the flat country enveloped for many miles together in thick woods. In vain he expects, as he moves on, to meet with some variety, some circumstance, connected with the happiness or occupation of man, to interest his feelings. A gloomy stillness and solitude prevail over the whole country. The silence of the forest is seldom interrupted, except by the cooing of wood-pigeons, or the rustling of wild animals, the only inhabitants of the deep jungles. The greatest degree of stillness prevails during the period about noon, when all classes of animals seek shelter from the ardent heat of the sun in the shady recesses of the woods." P. 3.

There are no natural lakes in the upland portion of the Kandyan country; but during heavy rains the rivers overflow their banks, and inundations of the plains take place to considerable extent. The superfluous water is eventually drained off by the Monsoon winds.

The high hills of the interior intercept the clouds during both Monsoons—hence the frequent genial showers which fertilise the soil and promote the exuberant foliage. At the change of the Monsoons there are heavy fogs in the mornings, which hover over the chains and vallies between the higher mountains, long after the tops of the lower hills become clear. Where the soil is clayey, the surface becomes hardened and divided by deep fissures, after drought of any continuance. There is a mystery, our author thinks, attending the hot land winds on this island. In some places, as on the Coromandel coast, they may be accounted for by the large tracts of heated sands over which they pass; but this cannot be their origin, he imagines, in Ceylon, for here they commence shortly after the fall of the rains, at the setting in of the S. W. Monsoons, and blow towards the coast over hills covered to their summits with trees, and over swampy vallies, thickly overgrown with low underwood, which extend to the very edge of the sea. Mr. Marshall must know that this is the season for the hot land winds along the Coromandel coast, and as the winds blow nearly from West to

East at this season, they must pass over the Continent in the neighbourhood of Cape Comorin, where they may become heated before they arrive at, and blow over Ceylon. Copious thermometrical tables follow, by which we observe that the mean temperature throughout the year, by night and by day, at Trincomalle, is somewhat above 80° Fahrenheit.

We must pass entirely over those portions of the work which contain accounts of the natural history, manners, customs, habits, food, occupations, &c. of the Candyan inhabitants, as not coming within the scope of this Journal. These details will prove exceedingly interesting to the traveller who visits Ceylon.

From the 3d chapter, respecting the prevailing diseases among the *native* inhabitants, we shall extract a few particulars. These diseases are principally climatorial—"few arising from the vitiated secretions of the living human body." Excepting small-pox, they suffer little from the specific contagions. Fevers of an intermitting and remitting type, and dysenteries, are the prevailing maladies.

> "The inhabitants observe, that hot, dry, and parching weather frequently precedes and accompanies the prevalence of fever. Partial showers, succeeding a long period of dry weather, are supposed to aggravate the influence of the cause of fever." 39.

This corresponds with the observations of Dr. Ferguson and other writers on hot climates. The provinces on the Eastern side of the interior terrace or high ground, are peculiarly insalubrious. Dysenteries sometimes arise idiopathically, but are more usually the sequelæ of fever, and are accompanied by an anasarcous torpid state of the system. Our author saw a number of Kandyans suffering from a wide-spreading ulcer, of the Phagedenic kind, which discharges a glairy fluid that dries into an elevated hard gray scab. It invades almost every part of the body, excepting the scalp, and is deemed by the natives incurable. It did not appear connected with any derangement of the digestive organs.

> "I have seen eight or ten cases treated with the blue pill, which was given so as slightly to affect the mouth. The symptoms were universally improved: indeed all those who took the medicine regularly recovered." 43.

The Kandyan medical works mention a cutaneous disease, consisting of seven varieties, and termed "*parangy lede*," sometimes "*rata waha sede*," or "foreign virulent disease," which our author imagines to be syphilis imported into Ceylon by the Portuguese; but there appears little to support the conjecture. The native doctors try simple remedies, for two or three months, and these failing, they universally have recourse to mercury.

Parturition does not appear quicker in Ceylon than in colder latitudes; but it is much safer in the result. Midwives assured our author that they have not witnessed a fatal case in a thousand parturient females. Flooding to a dangerous extent is unknown. When the superior extremity presents they endeavour to return it. One midwife informed Mr. M. that, in two instances, after failing to return the arm, she introduced her hand into the womb, grasped the child by the feet, and in this manner delivered her patient.

The author next presents us with a long account of *native medicine*, occupying 25 pages of letter-press. It is a tissue of ignorance, error, and superstition. Matters of mere curiosity, totally destitute of all utility, should be introduced with great caution into medical writings. Nevertheless this portion of the work will be read by the European medical officer, while lolling on his couch beneath the fervour of a Ceylonese sky, as a poor substitute for a Scotch novel, a quarterly review, or a monthly magazine. In this country we cannot throw away *time* on such absurdities—*time* being a scarce and valuable article here, but a heavy drug in the Indian market.

The second part of Mr. Marshall's work opens with an account of the physical constitution, and moral habits of the various classes of troops, Europeans, Malays, Caffries, and Indians, at the different stations in Ceylon. From this portion we shall cull a few particulars that may prove, more or less, interesting to our readers.

Our author observes that Europeans, on their arrival in Ceylon, generally undergo some change (and that not for the better) in their corporeal and mental functions. Their constitutions become irritable, and easily affected by stimuli. Many of them experience some degree of emaciation. The skin loses the ruddy hue of robust health, and assumes a pale yellowish shade. Moderate exercise becomes fatiguing, and the mind is indisposed to much application. Some individuals, however, of the higher classes, who are subjected to little fatigue, live temperately, and do not expose themselves much to the direct rays of the sun, are not greatly liable to acute diseases; but no care can entirely prevent the debilitating influence of the climate, and a tendency to chronic disease of some of the internal organs. Senescence frequently precedes old age at a considerable distance. Even females, who of course are little exposed, soon lose the plumpness of health—the countenance becomes sallow—the general complexion pale and colourless.

The fatal diseases to which Europeans are subject in Ceylon, are fevers, abscesses in the liver, and dysentery; but they are exempted, or nearly so, from a long catalogue of other diseases. Intemperance, in drink, is a common failing among British soldiers in all countries—in warm climates it is particularly injurious. In many parts of Ceylon Arrack may be procured for about sixpence a quart—the temptation, therefore, is seldom resisted.

The MALAY troops make good soldiers when properly managed. Their principal food is rice, dressed as throughout India, with spices, and sometimes *ghee* or butter. Those who have been born in Java and the neighbouring isles have a great propensity towards smoaking and eating opium. The habit once acquired can never be broken. Like intemperance in drink, it destroys the mental and physical powers in the end. Malays are liable to pectoral complaints, particularly pneumonia, and also phthisis and asthma. They are likewise affected with endemic fever when exposed to its causes—chiefly in the form of intermittents. To scabies they are greatly liable. The females are distinguished for fecundity—the children thrive remarkably well, and eventually contribute to recruit the regiment.

Not so with the AFRICANS, or CAFFRIES, as they are called. They are very liable to cachexia, phthisis, and enlargement of the lymphatic glands; but are almost wholly insusceptible of the endemic fevers of the country.

"Ceylon appears to have been extremely unfavourable to the health and propagation of Caffries. Not a trace of the many thousands brought to it by the Portuguese colonial government is to be perceived. The same may be said of a colony of Africans, which was imported about the year 1782, by Governor Van de Graaf." P. 80.

This high degree of mortality among the offspring of Caffries, (for they generally wither and die before they reach their 14th year,) cannot be ascribed either to neglect on the part of their parents, or to their being exposed to great hardships. The mothers are very attentive to their children, and the Caffry families are liable to no inconvenience, except when moving from one station to another. It is curious that the Caffrie children, by indigenous mothers, thrive as badly as the pure descendants of Africans.

The diseases of the *native Indian troops* are comparatively simple. Intermittent fevers, inflammation of the lungs and of the intestines, are the principal. These troops have little fortitude under disease, and their physical frames seem frequently to possess but a very moderate share of the principle of resistance to the inroads of disease, and of the powers of renovation.

Our author next introduces useful and valuable remarks on the medical topography of the individual stations, which, of course, we shall pass over, as well as the succeeding chapter on "the employment and health of the troops, from 1815 to 1820, with annual tables of the mortality, &c."

The third part of the work before us contains "Brief Remarks on the prevailing Diseases," beginning with FEVERS. These were of the remittent and intermittent types. We need not go over again for the hundred thousandth time, the symptoms of remittent fever. Most of those who died were opened.

"In cases where the disease terminated rapidly, there were very seldom any remarkable changes of structure observed. Even when the progress had been considerably protracted, many cases occurred where the structural derangement was apparently of little importance. The morbid structure discovered on dissection was rarely of such a degree as to appear to be the immediate cause of death." 139.

The more common changes of structure in the brain were, serous effusion under the dura mater, or between the arachnoid and pia matral tissue, increased vascularity of the brain and membranes, and serous effusion in the ventricles. In the thorax, portions of the lungs were occasionally found gorged with blood. In the abdomen, the liver frequently evinced a deviation from healthy structure. "Sometimes it was unusually red, at other times the colour was darker than natural; occasionally the organ appeared gorged with blood, and sometimes it seemed to contain less of that fluid than usual." The bile was unnatural in appearance—sometimes brown or coffee-coloured, and often resembling pitch in colour and consistence. The villous coat of the large intestines was sometimes found dark red and pulpy—occasionally incipient mortification had taken place. The spleen was often found enlarged.

The treatment employed by the medical officers was commonly antiphlogistic. In the early stage of the disease venesection was generally resorted to, particularly if there was much reaction. Cathartics were regularly administered, and febrile heat was reduced by the affusion of cold water.

"The early and repeated use of the lancet seldom failed to abate the violence of the most urgent symptoms of fever. Frequently, however, although the symptoms were meliorated, a fatal issue of the disease could not be averted. There are some grades of tropical fever which appear to be almost beyond the power of medicine." P. 143.

When the bleeding, purging, and cold affusions failed to arrest the progress of the disease, our author could only stand by, "and obviate occasional symptoms."

In the first case detailed, at page 144, we were somewhat surprized to find it seriously stated that there were thirty minims of serous effusion in each ventricle, and an equal quantity at the base of the brain. Thirty minims of water would not wet the base of the brain, so that it is pathological trifling to talk of such an effusion.

In the chapter headed "Inflammation of the Liver," Mr. Marshall observes that he has seen no reason to believe that the temperature of a tropical climate acts at all as a spur on the secretory function of the liver, increasing the formation of bile. But he offers no facts or observations to support his own opinion, or invalidate the contrary one entertained by almost every medical man who has visited the torrid zone. We do not deem it necessary therefore to enter into any argument on the subject. This chapter contains some good observations, but none of them new, on hepatic inflammation, as it occurs between the tropics. His practice is solely depletory by bleeding and purging.

"Should the acute symptoms subside without a return of health, and should there be no manifest proofs of the formation of pus in the liver, the use of mercury may then be tried, together with frequent moderate purgation. A mild degree of salivation is sometimes useful in this stage of the disease. When the liver contains an abscess, I suspect no quantity of mercury will cause ptyalism. Under such circumstances, the exhibition of mercury frequently occasions a soreness and heat of the gums, but rarely, if ever, ptyalism. Do other states of structural derangement of the liver (such as induration, or morbid softness) prevent the system from yielding to the salivatory operation of mercury?" 160.

As far as our observations extend there is peculiar difficulty in affecting the system with mercury in all organic diseases of the liver. The reason we cannot divine.

In the chapter on "a particular species of palsy," our author brings forward some curious observations which he had an opportunity of making in the year 1812, several cases of this disease having then occurred in the 4th Ceylon regiment.

For the most part, the complaint commenced with pain in the muscles of the thighs and legs, particularly the bellies of the gastrocnemii, accompanied by a general numbness of the extremity and imperfect power of loco-motion. A sensation as if hot water or sand were running over the parts is sometimes felt—at other times, a sense of formication. The appetite is seldom much affected during the early stage of the disease.

" When the disease has made considerable progress, the patient is unable to walk steadily. Standing or walking, in general, greatly aggravates the uneasiness of the limbs. The patients have an infirm tottering gait, and those whose hands are affected lose the power of feeding themselves. They seldom enjoy sound sleep, although they seem to labour under a sluggish inactivity and an unwillingness to exert themselves." 161.

The progress of the disease is sometimes protracted to several months; and in the advance of the complaint the patients express their sensations of the affected parts to be as if they were dry or dead, and almost entirely without feeling. Loss of appetite, indigestion, and emaciation soon follow—the limbs lose their natural temperature—the extensor muscles become paralytic, while the flexors seem still to have some force, thus contracting the joints. The pulse is frequent, thready, and fluttering—the vital functions greatly impaired, and eventually death ensues. The above is a description of the disease in its most violent form; but there are many gradations of severity. It is not met with among the indigenous inhabitants of the island. It is chiefly found among the Caffries.

" Europeans are not exempt from a similar kind of paralysis. In them the disease invades more suddenly than among Africans. A European artillery-man stationed at Tingall slept all night in a house, the doors and windows of which remained open. Next morning, on attempting to get out of bed, he fell precipitately to the ground. He had completely lost the voluntary power of moving his inferior extremities: his legs were cold, benumbed, and but little sensible to external impressions. By the application of warmth and the constant use of frictions, some relief was soon obtained. Five weeks after the supervention of the disease in his legs and feet, his hands became affected. Frictions and warm clothing were adopted, and seemed to be useful. When six months after the commencement of the disease had elapsed, he had not entirely regained the use of his hands. He was then unable to hold a small object with any degree of firmness between his fingers. His feet were less warm than natural: he complained that they felt numbed and torpid. He could walk pretty well on a level road, but ascending or descending a hill gave him great uneasiness. During exercise he regained in part the sensibility of his feet, but the advantages of exertion were only temporary." 163.

The most apparent causes of this disease are the application of cold and moisture to the body, especially when asleep —intoxication—violent exercise in the sun—insolation when sleeping—suddenly obstructed perspiration—and long fasting.

" The indication of cure seemed to be to stimulate the general system, and to excite the circulation of the blood in the extremities. These appeared to be best fulfilled by an improved diet, friction of the affected extremities, warm bath, fomentations, moderate exercise, warm clothing, &c. Mild cases were, for the most part, much benefited by this treatment. Some, however, resisted every mode of cure. In these cases the symptoms became gradually more aggravated and less under the influence of medicine. Severe cases rarely recovered." 164.

The fourth chapter is on dysentery. In the author's symptomatology of this well-known disease, we cannot expect to find any thing new. The following passage exhibits our author's *ratio symptomatum* and indeed pathology of dysentery—to many parts of which Mr. Marshall cannot lay claim, in point of originality.

"The following seems to be the progress of the local symptoms of dysentery. During the early stage, it may be presumed that the capillary arteries of the villous coat of the large intestines are in a state of active congestion. This state is evidenced by an increase of the natural mucous secretion, more or less commixed with blood. As the disease advances, fluids are effused into the coats of the intestines, more particularly into the villous coat. The coats of the intestine become thickened, unequal, and the cavity contracted. The villous coat is now covered with a slimy muco-purulent substance: which, mixed with blood, effused from ruptured blood-vessels, forms the discharges passed during a considerable period of the disease. Patches of the villous coat eventually slough. The sloughs are commonly dark-coloured, and have a grumous appearance. The gangrene extends, and death, in some cases, does not take place until large portions of the villous coat, and sometimes of a part of the other coats of the intestine, sphacelate. Under the gangrenous stage of the disease, the evacuations are fluid, brown-coloured, and excessively offensive: they seem to consist chiefly of a thin fetid serum." 175.

In the foregoing pathology the local symptoms are too much confined to the mucous membrane of the large intestines, and indeed the ratio symptomatum altogether is too confined. The author does not extend his views to the deranged functions of the skin and of the liver—derangements which are to be kept in mind, not only in a pathological but a therapeutical point of view.

For the cure of this formidable disease, our author seems to depend chiefly on venesection, a natural consequence of his considering inflammation of the mucous membrane of the intestines as the cause of the disease. But Mr. Marshall observes, or rather confesses, that "the influence of blood-letting over inflammation of the mucous coat of the intestines is often very feeble." Upon mild purgatives, fomentations to the belly, the warm bath, blisters, opiates, injections, our author seems to rely, after venesection. When the intestines are disorganized, then, he says, we may try mercury, given to ptyalism. Now we would absolutely forbid the use of mercury under such circumstances. But we would exhibit it *early*, and before any disease of structure took place, as a most valuable auxiliary to local and general bleeding, antimony, and the other means before mentioned. Mr. Marshall seems to have no idea of mercury being an important mean of cure in tropical dysentery, and, therefore, we shall extract from pages 184 and 185, a report of Dr. Paterson, which bears on the point in question.

"Dr. Paterson, Assistant-surgeon, 45th regiment, has much confidence in the liberal use of mercury, combined with copious venesection. He has had considerable experience in the treatment of Europeans under dysentery; and, as he is an intelligent and attentive medical officer, his sentiments demand our regard.

"In all cases of dysentery, which come early under his care, with much vascular commotion, he bleeds freely. Five or six pounds are sometimes taken from the arm in the course of two or three days. He thinks himself warranted in repeating venesection until the blood drawn has no buffy coat, the pain be much relieved, and the blood passed by stool greatly diminished. He gives occasional purgatives, such as small doses of neutral salts, during the course of the disease. With respect to the exhibition of mercury, the following are his views: 'In the cure of dysentery,' he says, 'my object is to bring the system under the influence of mercury as speedily as possible; and, with this view, a scruple of calomel is given morning and evening, having previously prescribed a laxative. The calomel seldom produces any inconvenience. In some cases, however, it is necessary, now and then, to interpose a dose of castor oil, or of some neutral salt, from its producing a degree of uneasiness in the bowels, and sometimes an increase of the tenesmus. After three or four doses of the calomel, the mouth becomes sore with ptyalism; and from this event, the disease uniformly yields. The cases of relapse, or long standing, however, receive no benefit from the mercury, as the mouth never can be properly affected. Instead of ptyalism, the mouth becomes hot and dry, with considerable irritation of the constitution, and, I think, a hurtful influence on the disease. From the insusceptibility of the system to the salivatory influence of mercury, I am induced to believe, that the disease has already produced such derangement of the structure of the intestines, as to render it doubtful if any medical treatment whatever could effect a cure; and from this event alone I generally pronounce an unfavourable prognosis.

"'I am far from pretending to say that mercury given in this way will uniformly cure dysentery; but in giving it a preference in the disease, as it appears in Kandy, I think I am warranted by its success in the hospital under my charge.

" 'Sudorifics, opium, baths, blisters, astringents, and enemata: with respect to these medicines, as means of cure, I possess little confidence. In most cases, however, I have recourse to them, from the temporary relief which they afford. The subjoined return will show the result of my practice in dysentery for nearly eight months, in Kandy, namely, from the 3d April to the 13th November 1820. A few of the cases, included in the return, had previously suffered under dysentery at Trincomale. Most of the cases were, however, primary attacks of the disease.

| Admitted. | Discharged. | Died. | Remaining. | |
|---|---|---|---|---|
| " 70 | 57 | 7 | 6.' " | 185. |

This return, Mr. Marshall allows, exhibits a smaller ratio of mortality than usually occurs among Europeans attacked with dysentery in Ceylon; and we humbly conceive that such facts should have induced both him and the Inspector of Hospitals, to give trial to a measure which has been recommended by such able observers in other parts of the tropics, in both hemispheres.

The remaining short Chapters, on Tetanus, Berriberri, Cholera, and Ulcers, present nothing particularly interesting to European readers, and, therefore, we shall pass them over. Upon the whole, Mr. Marshall's work, though it somewhat disappointed us in certain parts, is creditable to his talents as an observant and intelligent surgeon. We recommend the work to every young surgeon about to embark for our East Indian possessions.

